# Immunogenomic analysis of concurrent lung cancer and tuberculosis reveals distinct immune milieu

**DOI:** 10.1186/s43556-025-00316-0

**Published:** 2025-10-31

**Authors:** Xiaoling Xu, Chaohui Bao, Nathaniel Deboever, Da Chen, Tianxiang Wang, Mara Antonoff, Yaping Xu, Yun Fan, Jianjun Zhang, Weimin Mao

**Affiliations:** 1 Department of Radiation Oncology, Shanghai Pulmonary Hospital, Tongji University School of Medicine, Shanghai, 200433 China; 2 Shanghai Institute of Hematology, State Key Laboratory of Medical Genomics, National Research Center for Translational Medicine at Shanghai, Ruijin Hospital, Shanghai Jiao Tong University School of Medicine, Shanghai, 200025 China; 3 Department of Thoracic and Cardiovascular Surgery, University of Texas MD Anderson Cancer Center, Houston, TX USA; 4 Department of Thoracic Surgery, Zhejiang Cancer Hospital, Hangzhou, China; 5 Department of Thoracic Surgery, Affiliated Hangzhou Chest Hospital, Zhejiang University School of Medicine, Hangzhou, China; 6 Department of Thoracic Oncology, Zhejiang Cancer Hospital, Hangzhou, Zhejiang 310022 China; 7 Department of Thoracic/Head and Neck Medical Oncology, University of Texas MD Anderson Cancer Center, Houston, TX USA

## Dear Editor,

Tuberculosis (TB) is associated with lung cancer (LC) carcinogenesis in up to 10% of patients. TB and its treatment adversely affect the anti-tumor immune environment. The immunosuppressive state involves complex mechanisms, including the upregulation of CD84 and the downregulation of interferon-gamma [1]. Therapeutic vulnerability and mortality in these patients depend on dynamic immune changes resulting from pathological or treatment-induced decay [2]. These alterations in immunity significantly impact the efficacy of immune checkpoint inhibitors (ICI), which are a cornerstone of LC treatment [3].

Regional variations in TB prevalence necessitate the establishment of local guidelines for detecting asymptomatic TB in lung cancer (LC) patients. This is crucial for understanding the distinct molecular and immune features associated with prolonged TB infection, which may influence responses to therapy [4]. Despite recent research, the tumor microenvironment (TME) in concurrent LC and TB remains poorly characterized. This study investigates the impact of TB on the LC TME and elucidates the associated host immune response. Additionally, we characterize non-tumor pulmonary tissue in LC and TB patients through integrated genomic and immune microenvironment analysis.

**Fig. 1:**
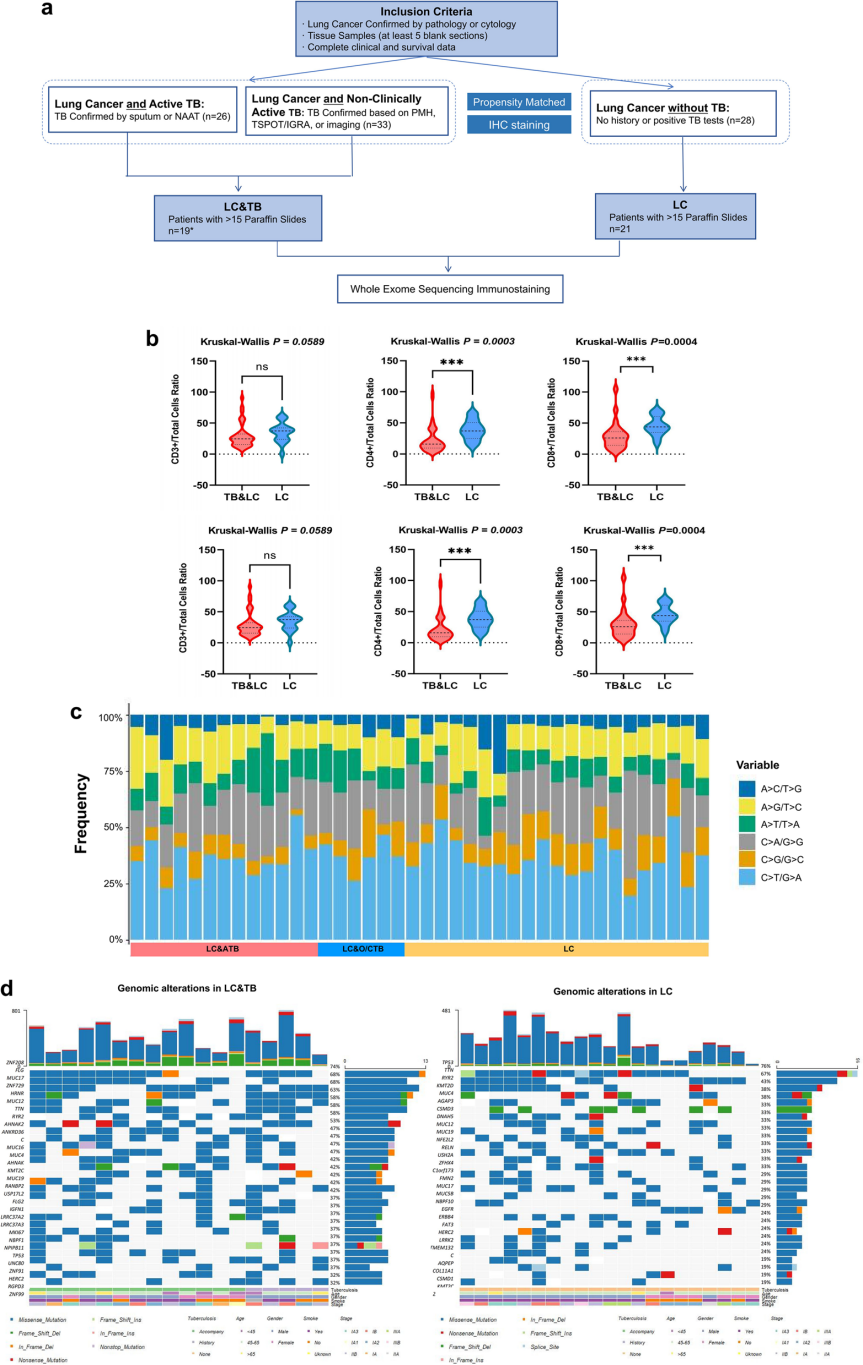
Comparative multi-omic profiling of lung cancer with vs. without tuberculosis co-infection. **a** Consort diagraph representing the inclusion process. Whole-exome sequencing (WES) was successfully performed on all 40 eligible patients-19 with combined lung cancer and tuberculosis (LC&TB) tumors and 21 with lung cancer (LC)-only tumors. **b** Correlations between tumor immune microenvironment factors and overall survival. The LC&TB group was compared with the LC group, by PD-L1 and CD8 expression, PD-L1 and CD4 expression, and PD-L1 and CD3 expression. The infiltration of CD3 +, CD4 +, CD8 + cells (number/mm2) were compared with LC&TB and the LC group. The ratio of CD3 +, CD4 +, CD8 + cells vs total cells were also compared. **c** Frequencies of mutation substitution types (classified as 6 substitution classes) in the genome among all samples. Vertical bars indicate individual patients, while vertical axis shows the frequency of each mutation category for a specific mutation type. Most patients with LC predominantly had C > T/G > A transitions. **d** Genetic alterations, types of substitution mutations, and gains/losses (CNVs) in patients with LC&TB or LC. The top 30 genomic alterations in LC&TB and LC patients. Vertical bars indicate individual patients, while vertical axis shows the frequency of each mutation category for a specific mutation type. Most patients with LC predominantly had C > T/G > A transitions. Abbrev: TB: tuberculosis, NAAT: Nucleic Acid Amplification Test, PMH: past medical history, TSPOT: T-cell spot test, IGRA: Interferon gamma release assay

The databases of three centers (Zhejiang Cancer Hospital, Shanghai Pulmonary Hospital, and Affiliated Hangzhou Chest Hospital) were reviewed for patients diagnosed with both LC and TB between 2013 and 2019 (Fig. 1a). LC diagnosis in all patients was confirmed by pathology and/or cytology. Active TB (ATB) diagnosis was defined as having three positive sputum-based Mycobacterium cultures or nucleic acid amplification tests (NAAT). Patients were also categorized as having TB if a positive culture or NAAT originated from the lung biopsy sample. Patients were classified as having non-clinically active tuberculosis (NCATB) based on medical records indicating a previous tuberculosis infection treated with a full course of pharmacotherapy, imaging results, and T-cell spot test (TSPOT) or interferon gamma release assay (IGRA) outcomes. For molecular and immune analyses, a control group was selected from the same databases, consisting of individuals without any tuberculosis diagnosis (LC). This control group was propensity-matched to the LC&TB group according to sex, age, smoking status, distant metastasis, and pathological lung cancer type (adenocarcinoma or non-adenocarcinoma) for molecular and immune profiling.

The samples analyzed in this study encompassed TME, with a focus on T cells within lung cancer tumors, both with and without tuberculosis. When available, the immunologic milieu of tuberculosis tissue samples was also examined. The TME of matched tumors with lung cancer only was investigated using the same markers as the control group. PD-L1 expression, along with tumor-infiltrating CD3 + T-cells, CD4 + T-cells, and CD8 + T-cells, was evaluated through immunohistochemistry (IHC). Tumors from both the LC&TB and LC cohorts with adequate materials (> 15 unstained slides) underwent DNA extraction and whole-exome sequencing (WES).

To investigate the molecular and immune characteristics of LC&TB tumors, a total of 2,147 patients were screened, and 59 patients with concurrent LC&TB and sufficient tumor tissues were identified, comprising 33 with ATB and 26 with NCATB. The majority of the cohort were male (*n* = 48, 81.4%) and smokers (*n* = 40, 67.8%), with a median age of 62 years (interquartile range [IQR]: 51–79). Most patients exhibited non-adenocarcinoma histology (*n* = 31, 52.5%), with a predominance of early-stage tumors (N0: *n* = 33, 57.9%) without distant metastasis (M = 0: *n* = 44, 75.9%). Additionally, we identified 28 patients with lung cancer without tuberculosis (LC group), who were propensity-matched for age, gender, smoking status, and histology, with available tumor tissues serving as controls for the LC&TB group.

To understand the TME in LC&TB, we conducted IHC using antibodies against PD-L1, CD3, CD4, and CD8 on LC&TB tumors and LC-only controls. The results demonstrated that LC&TB tumors exhibited significantly lower PD-L1 expression and reduced infiltration of CD3 +, CD4 +, and CD8 + T cells compared to their LC-only counterparts (Fig. 1b). Additionally, LC&TB tumors were significantly enriched in PD-L1-/CD3- tumors. No significant differences were observed between tumors with ATB and NCATB. Among the TB&LC group, ten patients had sufficient material from both tumor samples and TB samples for immune profiling. Due to the small sample size, no significant differences were detected; however, TB tissues showed numerically higher infiltration of CD3 + (average 15% versus 6%) and CD4 + (16% versus 0%) T cells compared to the tumor samples. Collectively, these findings indicate a cold TME in LC&TB tumors.

Subsequently, we utilized WES on LC&TB tumors (*n* = 19) and their LC-only counterparts (*n* = 21) with adequate material available to investigate the genomic characteristics associated with the cold TME in LC&TB tumors. The predominant base substitution observed was C > T in both the LC&TB and LC groups, with frequencies of 34.9% and 35.27% (Fig. 1c), respectively. The most frequently mutated genes in these patients included TTN, TP53, and MUC17 (Fig. 1d). Notably, TP53 mutations were significantly more prevalent in the LC&TB group compared to the LC group (*p* = 0.003). No significant differences were found in other cancer-associated genes such as EGFR, PIK3CA, KRAS, BRAF, or CDKN2A (all *p* > 0.05).

Importantly, LC&TB tumors exhibited a significantly higher TMB compared to LC tumors (18.76 mutations/Mb vs 10.45 mutations/Mb, *p* < 0.001). Consequently, the predicted neoantigens were significantly elevated in LC&TB tumors (1,017.21 vs 395.29, *p* < 0.001). As anticipated, the number of nonsynonymous mutations correlated with the number of neoantigens (*r* = 0.74, *p* < 0.01). Furthermore, PD-L1 expression was not associated with TMB (*r* = 0.71, *p* = 0.258) or TP53 mutations (*p* = 0.027).

LC&TB tumors exhibited a higher frequency of copy number aberrations and an increased incidence of IFN-γ pathway gene loss. Despite presenting with a high TMB and a greater prevalence of TP53 mutations—both of which are generally associated with a more active anti-tumor immune response—LC&TB tumors were characterized by a colder TME [5]. To investigate this phenomenon, we analyzed the copy number variations (CNV) in the LC&TB cohort compared to the LC cohort. In the LC&TB group, the median number of CNVs was 241 per tumor (IQR 177–320), significantly surpassing the median of 160 (IQR 108–194) observed in the LC group (*p* = 0.002). Notably, the loss of IFN-γ pathway genes was numerically more prevalent in LC&TB tumors than in LC tumors, and a positive correlation was identified between the CNV burden and the burden of copy number loss of IFN-γ pathway genes.

In conclusion, our genomic and immune profiling study of 59 patients with LC&TB demonstrated an overall cold TME, despite the presence of high TMB and neoantigen burden. These tumors exhibited a substantial burden of CNVs and a heightened frequency of IFN-γ pathway gene loss, which may contribute to immune evasion in LC&TB tumors. Future research, particularly through multi-institutional collaborations involving larger patient cohorts receiving immunotherapy, is essential to comprehensively elucidate the distinct molecular and immune characteristics of this unique patient population. This understanding will enhance insights into the mechanisms underlying lung cancer carcinogenesis in this demographic and the effects of TB infection on immunotherapy outcomes. Ultimately, this knowledge will aid in the development of personalized therapeutic strategies aimed at preventing lung cancer in TB patients and effectively treating those with concurrent TB and lung cancer.

## Supplementary Information

Supplementary Material 1.

## Data Availability

The cBioPortal database was used to evaluate the relationships between survival outcomes and gene mutation frequency, smoking status, and tumor mutational burden (TMB). The relationships between TIL characteristics and mutations in TP53 were evaluated using the TIMER database (versions 1 and 2, http://timer.cistrome.org/).
