# Supplementary material for: Immunogenomic analysis of concurrent lung cancer and tuberculosis reveals distinct immune milieu
Source: Mol Biomed. 2025 Oct 31;6:93. doi: 10.1186/s43556-025-00316-0 (PMC12575902; doi:10.1186/s43556-025-00316-0)
Supplement: Supplementary file 1 — Supplementary Material 1. [file 43556_2025_316_MOESM1_ESM.docx]

**Immunogenomic analysis of concurrent lung cancer and tuberculosis reveals distinct immune milieu**

Xiaoling Xu^1*^, Chaohui Bao^2*^, Nathaniel Deboever^3^^*^, Da Chen^4*^, Tianxiang Wang^5^, Mara Antonoff^3^, Yaping Xu^1**^, Yun Fan^6**^, Jianjun Zhang^7**^, Weimin Mao^4**^

^1^ Department of Radiation Oncology, Shanghai Pulmonary Hospital, Tongji University School of Medicine, Shanghai 200433, China

^2^Shanghai Institute of Hematology, State Key Laboratory of Medical Genomics, National Research Center for Translational Medicine at Shanghai, Ruijin Hospital, Shanghai Jiao Tong University School of Medicine, Shanghai 200025, China.

^3^ Department of Thoracic and Cardiovascular Surgery, University of Texas MD Anderson Cancer Center, Houston, TX

^4^ Department of Thoracic Surgery, Zhejiang Cancer Hospital, Hangzhou, China

^5^ Department of Thoracic Surgery, Affiliated Hangzhou Chest Hospital, Zhejiang University School of Medicine, Hangzhou, China

^6^ Department of Thoracic oncology, Zhejiang Cancer Hospital, Hangzhou, China

^7^ Department of Thoracic/Head and Neck Medical Oncology, University of Texas MD Anderson Cancer Center, Houston, TX

^*^Co-First Authors

**Co-Last Authors

**Correspondence to:**

Yaping Xu

Department of Radiation Oncology, Shanghai Pulmonary Hospital, Tongji University School of Medicine, Shanghai 200433, China

Email: xuyaping1357@163.com

Yun Fan, Department of Thoracic Oncology, Zhejiang Cancer Hospital, Hangzhou, Zhejiang 310022, China.

E-mail: fanyun1218@163.com

Jianjun Zhang

Department of Thoracic/Head and Neck Medical Oncology, University of Texas MD Anderson Cancer Center, Houston, TX

Email: JZhang20@mdanderson.org

Weimin Mao

Department of Thoracic Surgery, Zhejiang Cancer Hospital, Hangzhou, China

Email: maowm1318@163.com

**Supplemental material**

**Materials and Methods**

**Study Design and Patient Population**

The databases of three centers (Zhejiang Cancer Hospital, Shanghai Pulmonary Hospital, and Affiliated Hangzhou Chest Hospital) were reviewed for patients diagnosed with LC&TB between 2013 and 2019. LC diagnosis in all patients was confirmed by pathology and/or cytology. Active TB (ATB) diagnosis was defined as having 3 positive sputum based- Mycobacterium culture or nucleic acid amplification tests (NAAT). Patients were also categorized as having TB if positive culture or NAAT originated from the lung biopsy sample. Patients were considered to have non-clinically active TB (NCATB) based on medical records (previous TB infection with full course of pharmacotherapy) or imaging, in addition to T-cell spot test (TSPOT) results or interferon gamma release assay (IGRA). For molecular and immune analysis, a control group was selected from the same databases without any TB diagnosis (LC). This group was propensity matched to the LC&TB group according to sex, age, smoking status, distant metastasis and pathological LC type (adenocarcinoma or non-adenocarcinoma) for molecular and immune profiling **(Table 1)**. This study was approved by the Internal Review Board of Zhejiang Cancer Hospital, Shanghai Pulmonary Hospital, and Affiliated Hangzhou Chest Hospital.

**Table 1 Clinicopathological characteristics of lung cancer patients with or without tuberculosis.**

| Patient Characteristics | LC&TB group (N = 59) | LC group (N = 28) | *P* value |
| --- | --- | --- | --- |
| Sex |  |  | 0.295 |
| Male | 48 (81.4%) | 20 (71.4%) |  |
| Female | 11 (18.6%) | 8 (28.6%) |  |
| Median Age  (Range) | 62 (51-79) years | 62 (37-75) years |  |
| Smoking status |  |  | 0.995 |
| Non-smoker | 19 (32.2%) | 9 (32.1%) |  |
| Smoker | 40 (67.8%) | 19 (67.9%) |  |
| Histology |  |  | 0.688 |
| Adenocarcinoma | 28 (47.5%) | 12 (42.9%) |  |
| Non-adenocarcinoma | 31 (52.5%) | 16 (57.1%) |  |
| Pathologic T stage |  |  | 0.132 |
| T1+T2 | 40 (69.0%) | 22 (84.6%) |  |
| T3+T4 | 18 (31.0% | 4 (15.4%) |  |
| Lymph node metastasis | |  | 0.319 |
| N0 | 24 (42.1%) | 15 (53.6%) |  |
| N1-3 | 33 (57.9%) | 13 (46.4%) |  |
| Distant metastasis |  |  | 0.931 |
| M0 | 44 (75.9%) | 21 (75.0%) |  |
| M1 | 14 (24.1%) | 7 (25.0%) |  |

**Immune profiling**

The samples analyzed in this work encompassed the tumor microenvironment (TME) with a focus on T cells of lung cancer tumors with or without tuberculosis. When available, the immunologic milieu of TB tissue samples were also analyzed. The TME of matched tumors with LC only was investigated using the same markers as control. PD-L1 expression, as well as tumor-infiltrating CD3^+^ T-cells, CD4^+^ T-cells, and CD8^+^ T-cells were evaluated using immunohistochemistry (IHC). The tumor expression of PD-L1 was quantified by the tumor proportion score (TPS), which is based on the proportion of tumor cells with positive staining. The proportions of CD3/CD4/CD8 staining on lymphocytes were evaluated as the proportions of positive cells among all nucleated cells in the stromal compartments; a proportion of ≥25% was considered positive.

**Genomic Anal*ysis***

Tumors from the LC&TB and LC cohorts with sufficient materials (>15 unstained slides) were subjected to DNA extraction and whole-exome sequencing (WES). Briefly, DNA was extracted and fragmented from formalin-fixed paraffin-embedded (FFPE) samples following which sequencing was performed which generated 150-bp paired-end reads as previously described. CNV burden is defined as the total number of copy number variations detected in each tumor sample.

**Analysis of publically available data**

The cBioPortal database was used to evaluate the relationships between survival outcomes and gene mutation frequency, smoking status, and tumor mutational burden (TMB). The relationships between TIL characteristics and mutations in TP53 were evaluated using the TIMER database (versions 1 and 2, http://timer.cistrome.org/). The 25% cutoff for TIL positivity was based on the IASLC TIL scoring criteria.

**Statistical analysis**

All data were expressed as mean and standard deviation or number and percentage, unless otherwise specified. Comparative statistical tests were used based on data distribution, and whether the data was paired. Linear correlations were evaluated using Pearson’s correlation coefficient. Multivariable survival analyses were performed using the Cox proportional hazards model, and survival analyses were performed using the Kaplan-Meier method. The statistical analyses were performed in the R statistical environment (version 3.5.1; Boston, MA, USA) and SPSS (version 24; IBM Corp., Armonk NY, USA) while GraphPad Prism (version 8.4; GraphPad, San Diego, CA, USA) was used to create the stacked bar graphs. Differences were considered statistically significant at two-sided P-values of <0.05.
